# Serum YKL-40 Level Is Associated with the Chemotherapy Response and Prognosis of Patients with Small Cell Lung Cancer

**DOI:** 10.1371/journal.pone.0096384

**Published:** 2014-05-06

**Authors:** Chun-Hua Xu, Li -Ke Yu, Ke-Ke Hao

**Affiliations:** 1 First Department of Respiratory Medicine and Nanjing Chest Hospital, Nanjing, Jiangsu, China; 2 Clinical Center of Nanjing Respiratory Diseases, Nanjing, Jiangsu, China; Ottawa Hospital Research Institute, Canada

## Abstract

This study was to explore the association between the serum YKL-40 level and the clinical characteristics, the response to chemotherapy and prognosis in small cell lung cancer (SCLC). Serum YKL-40 levels were detected and compared in 120 patients with SCLC pre- and post-chemotherapy, and in 40 healthy controls. Receiver operating characteristics (ROC) curves were adopted for diagnosis and calculation of area under ROC curve in SCLC. The Kaplan–Meier method, univariate and multivariate Cox regression analysis were used to analyze the correlation between pre-chemotherapy serum YKL-40 levels and progression-free survival (PFS) and overall survival (OS). The pre-chemotherapy serum YKL-40 levels were significantly higher than those of the controls (*p*<0.001). The post-chemotherapy serum YKL-40 levels in the SCLC cases were lower than pre-chemotherapy serum YKL-40 levels in these cases (*p* = 0.026). The patients with high serum YKL-40 showed a poorer response to chemotherapy than those patients with low serumYKL-40 (*p* = 0.031). Univariate analysis revealed that SCLC patients with high serum YKL-40 had a shorter PFS and OS than those with low serum YKL-40 (HR of 1.74, *p* = 0.033; HR of 1.33, *p* = 0.001). Cox multivariate analysis indicated that YKL-40 was an independent prognostic indicator of PFS and OS (HR of 1.12, *p* = 0.029; HR of 1.84, *p* = 0.025). Kaplan–Meier survival curves further confirmed that patients with low serum YKL-40 have longer PFS and OS (*p* = 0.016 and *p* = 0.041, respectively). These results suggest that YKL-40 is a potential prognostic marker of chemotherapy response in SCLC.

## Introduction

Lung cancer is the leading cause of cancer-related death worldwide, with more than 1.2 million deaths each year [1]. Small cell lung cancer (SCLC) accounts for up to 15% of total lung malignancies [2]. Despite the often dramatic response to chemotherapy and radiotherapy in patients with SCLC, most die from recurrent disease [3]. With rapid tumor doubling rate and early metastasis, over half of SCLC patients present with extensive disease [4]. As a result, five years survival rate in SCLC is only 10%–26% [5].

Tumor markers play a key role in patient management for many malignancies. The potential uses of serum tumor markers include aiding early diagnosis, determining prognosis, predicting response or resistance to specific therapies, and monitoring therapy in patients with advanced disease. Tumor markers that are currently available for lung cancer, such as progastrin-releasing peptide (ProGRP), carcinoembryonic antigen (CEA), and neuron-specific enolase (NSE), are not satisfactory for diagnosis at an early stage or for monitoring the disease because of their relatively low sensitivity and specificity in detecting the presence of cancer cells [6]–[8]. Therefore, more studies are required to discover novel biomarkers in order to predict the chemotherapy response and improve the prognosis of patients with SCLC.

YKL-40 is produced by cancer cells and may play a role in cancer cell proliferation and angiogenesis [9]. Recent studies have shown that elevations of serum YKL-40 were reported in various malignant tissues, including breast cancer, gastric cancer, and ovarian cancer [10]–[13]. The association between increased YKL-40 and a poor prognosis has been well documented [11]–[17], including lung cancer [18]–[20]. However, the association between serum YKL-40 level and the clinical characteristics, especially the response to chemotherapy and prognosis in SCLC remain largely unknown.

The purpose of this study was to investigate the clinical role of serum YKL-40 in SCLC patients. Our aim is to evaluate the possibility of serum YKL-40 as a biomarker in SCLC.

## Materials and Methods

### Patients

The study included 120 SCLC patients, who were diagnosed at the Nanjing Chest Hospital between January 2007 and December 2011. Mean age of the patients was 64.5±5.6 years, 95 male and 25 female. All the patients underwent clinical examination, CT scans of the chest and brain, CT and ultrasonography of the upper abdomen, fibreoptic bronchoscopy, bone scanning, and positron emission tomography. Staging was carried out according to the veterans administration lung cancer group (VALG) staging system [21]: Limited disease (LD) was defined as disease confined to one hemithorax including the mediastinal lymph nodes and/or the supraclavicular lymph nodes; extensive disease (ED) was defined as having limited disease or malignant pleural effusion. Smoking history was obtained from the health interview questionnaire. Current and former smokers were classified as “smoker” and never smokers as “nonsmoker”. The control subjects consisted of 40 healthy volunteers (31 males and 9 females with a mean age of 57.6±10.8 years), as documented by a general health examination in the same period. They were not on any medication, and had no clinical signs or symptoms of cancer, liver, kidney or hormonal disease. The demographic features and clinical characteristics of the studied groups are illustrated in Table 1.

**Table 1:** Clinical characteristics of SCLC patients and controls.

Characteristics	SCLC (N = 120)	Control (N = 40)	*p*-value
Age, yr			
Range	46 78	45 74	
Mean	64.5±5.6	57.6±10.8	0.247
Gender			
Male	95 (79.17%)	31 (75.50%)	
Female	25 (20.83%)	9 (22.50%)	0.176
Smoking condition			
Smoker	80 (66.67%)	29 (72.50%)	
Nonsmoker	40 (33.33%)	11 (27.50%)	0.138

p values were calculated using chi-square test.

All SCLC patients received chemotherapies for a maximum of six cycles. Chemotherapy regimens are presented in Table 2. Computed tomography (CT) scans were performed after 2 cycles of chemotherapy. All patients had measurable disease. Response categories were defined according to the Response Evaluation Criteria in Solid Tumors (RECIST) as complete response (CR), partial response (PR), stable disease (SD) and progressive disease (PD) [22]. For data analysis, CR and PR were combined as response that was sensitive to chemotherapy, while SD and PD were grouped as non-response. Follow-up information was obtained by phone investigations. The median follow-up of surviving patients at the time of analysis was 14 months (range, 5–36 months). The date of the last follow-up was March 21, 2013. Progression-free survival (PFS) was defined as the time interval between the date of diagnosis and the date of disease progression or last follow-up. Overall survival (OS) was defined as the time interval between the date of diagnosis and the date of death or the last follow-up.

**Table 2:** The clinicopathological characteristics of SCLC and the association with serum YKL-40 levels.

Group	n	YKL-40 (ng/mL)	*p*-value
Age, yr			
<60	52	68.47±26.95	0.209
≥60	68	74.81±27.76	
Gender			
Male	95	72.85±26.94	0.541
Female	25	69.06±29.81	
Smoking status			
Nonsmoker	40	66.51±27.26	0.118
Smoker	80	74.84±27.33	
Performance status			
0, 1	86	73.92±26.52	0.265
2, 3	34	67.36±29.65	
Disease stage			
Limited	70	67.31±25.98	0.024*
Extended	50	78.71±28.39	
Chemotherapy regimen			
EP	102	70.67±25.62	0.376
IP	18	73.16±27.38	

EP, cisplatin with etoposide; IP, cisplatin with irinotecan.

*Significant difference.

The protocol was approved by the Ethics Committee of the Nanjing Chest Hospital, and written informed consent was obtained from all patients and healthy controls before the study. Doctors recorded the obtainment of the written consent in patients' clinical files. After the treat agreement was signed but prior to the chemotherapy, the patients also noted in the treat agreement that she/he was informed about and agreed to participate in this study. The Ethics Committee approved this written consent procedure and had unscheduled inspection of documents and records to assure the study was compliant.

### Serum Collection

Peripheral blood samples were obtained from the healthy controls and from the patients after diagnosis but prior to any treatments. For the 120 SCLC patients treated with chemotherapy, serum samples were obtained 3 weeks after completion of the second chemotherapy cycle. The whole blood samples were promptly centrifuged at 3000 rpm for 15 minutes and the supernatant stored at −80°C until use.

### Detection of Serum YKL-40 Levels

The serum levels of YKL-40 were measured using an YKL-40 ELISA kit ((Quidel, San Diego, CA, USA). A volume of 50 µl of 2-fold diluted samples was added to a 96-well plate covered with YKL-40 monoclonal antibody and incubated for 2 h at room temperature. After aspirating and washing each well four times, 50 µl of YKL-40 conjugate was added and the wells incubated for 2 h at room temperature. Each well was washed an additional four times to remove residual liquid and 200 µl of substrate solution was added to each well and the wells incubated for 30 min in darkness. After addition of stop solution, absorbance was measured at 450 nm on a Biotek-elx800 microplate reader (Roche, USA), and the YKL-40 concentration determined. The sensitivity of the ELISA was 20 ng/mL. All samples were measured in duplicate in two separate plates. For duplicate samples, intra-assay coefficient of variation of <5% and the inter-assay coefficient of variation of <6% were accepted.

### Statistical Analysis

Statistical analysis was carried out using SPSS 17.0 software. Data were expressed as mean ± standard deviation (SD). Continuous variables were compared using *t* test. Survival curves were plotted by the Kaplan-Meier method and compared using the log-rank test. Survival data were evaluated using univariate and multivariate Cox regression analysis. Receiver operating characteristics curves (ROC) were adopted to determine the diagnostic value of YKL-40 in cancer. For each ROC, an optimal cut-off point was determined as the value of the parameter that maximized the sum of specificity and sensitivity. A value of *p*<0.05 was considered significant.

## Results

### Pre-chemotherapy Serum YKL-40 Levels in the SCLC Patients and the Controls

**Figure 1:**
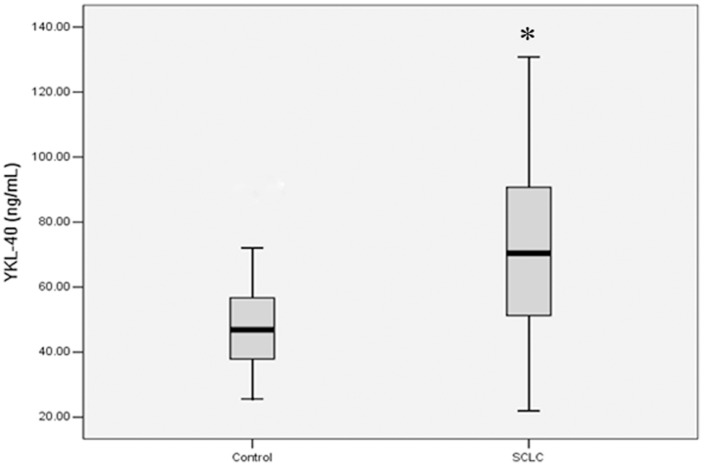
Pre-chemotherapy serum YKL-40 levels in SCLC patients and healthy controls. Among 120 SCLC patients, the serum levels of YKL-40 were (72.06±27.48) ng/mL, which were significantly higher than (48.41±13.63) ng/mL in healthy controls (*p*<0.001, *t*-test).

As shown in Figure 1, the mean level of YKL-40 in the pre-chemotherapy serum from SCLC patients (n = 120) was (72.06±27.48 ng/mL), which was significantly higher than the controls (n = 40, 48.41±13.63 ng/mL, *p*<0.001).

### Relationship between Pre-Chemotherapy Serum YKL-40 Levels and Clinicopathological Characteristics

Table 2 showed the pre-chemotherapy serum YKL-40 levels in SCLC with different clinicopathological characteristics. The serum YKL-40 levels were significantly correlated with disease stage (*p* = 0.024), while there were no difference with age (*p* = 0.209), gender (*p* = 0.541), smoking status (*p* = 0.118), performance status (*p* = 0.265), and chemotherapy regimen (*p* = 0.376).

### Diagnostic Value of Serum YKL-40 in SCLC

**Figure 2:**
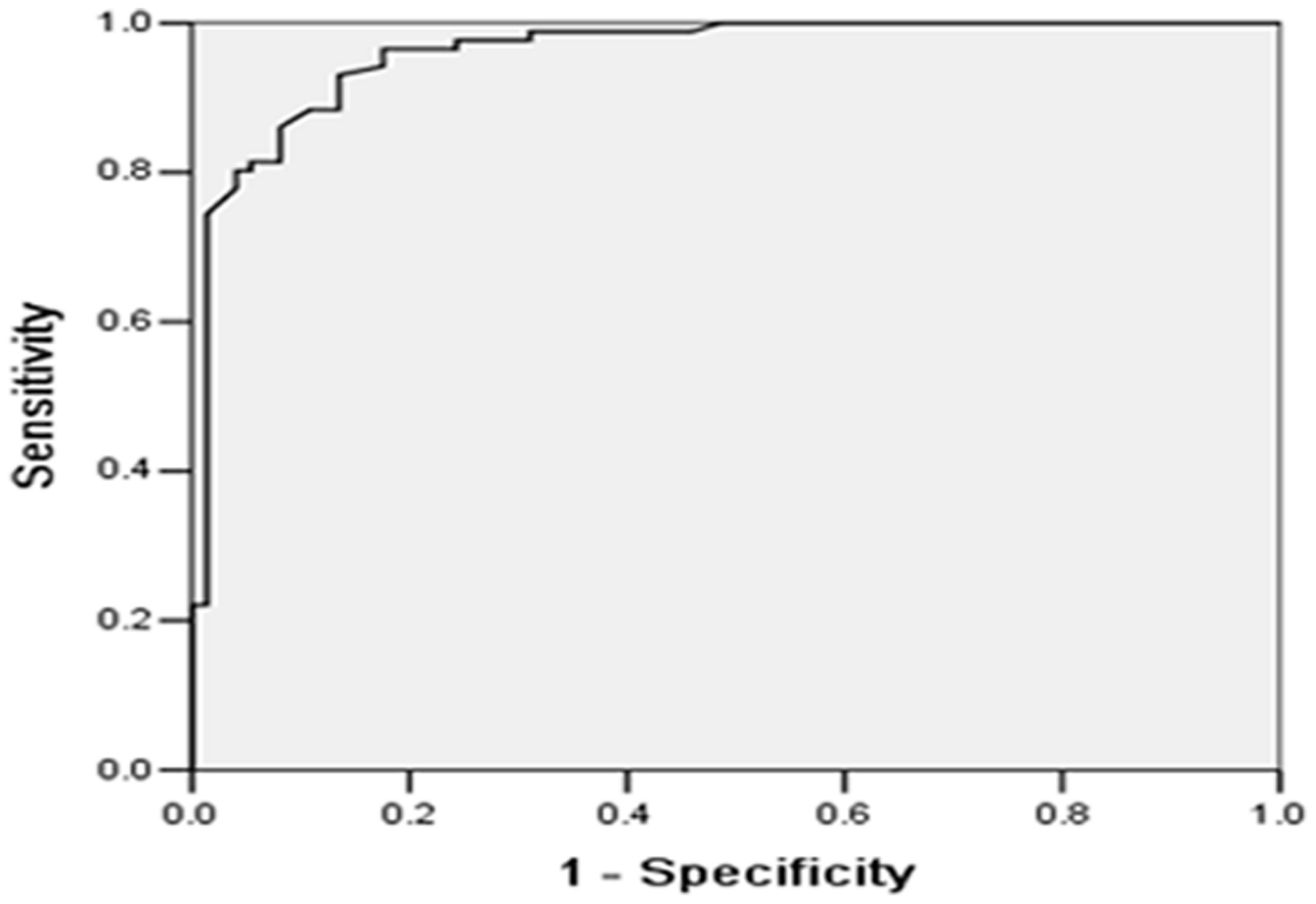
ROC curve of the serum YKL-40 levels of 120 SCLC patients and 40 controls. Serum levels of YKL-40 among 120 SCLC patients and 40 healthy controls were determined. The diagnostic potentials of YKL-40 were assessed by ROC curves. The AUC value was 0.96.

ROC curves were calculated based on the serum YKL-40 levels of the 120 SCLC patients and 40 controls (Figure 2). The estimated area under the ROC curve was 0.96 (95% CI (confidence interval), 0.93–0.99). The optimal cut-off value of serum YKL-40 level was 65.7 ng/mL (low vs. high) based on the maximization of the sum of specificity and sensitivity, resulting in 67.5% sensitivity, 95.0% specificity, and 74.4% accuracy for the diagnosis of SCLC.

### Serum YKL-40 and Response to Chemotherapy

**Figure 3:**
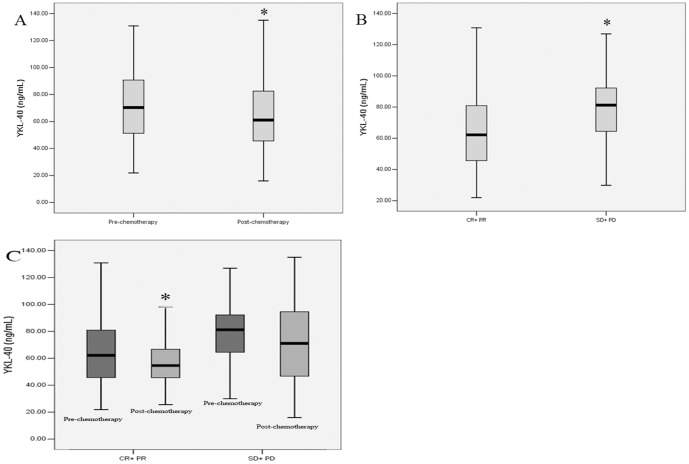
Distribution of serum YKL-40 levels. Serum YL-40 levels in the SCLC patients significantly decreased after chemotherapy (*p* = 0.026) (A). The pre-chemotherapy serum YKL-40 levels were lower in the sensitive to chemotherapy group than in the resistant to chemotherapy group (*p* = 0.004) (B). Serum YKL-40 of responders after chemotherapy were significantly decreased (*p* = 0.029), while YKL-40 of non-responders did not change significantly (*p* = 0.256) (C).

To determine the correlation between serum YKL-40 levels and chemotherapy response, the YKL-40 levels between pre- and post-chemotherapy were analyzed in SCLC patients who received 2 cycles of chemotherapy. Generally, serum YL-40 levels significantly decreased after chemotherapy (*p* = 0.026) (Figure 3A). Among 120 patients, 32 (26.67%) patients achieved CR, 33 (27.5%) patients achieved PR, 35 (29.17%) patients achieved SD, and 20 (16.67%) patients achieved PD. Of the 81 patients with high serum YKL-40, 38 (46.91%) responded to chemotherapy with either complete response, or partial remission. Of 39 low serumYKL-40 patients, 27 (69.23%) exhibited a response to chemotherapy. The difference in response to chemotherapy between high and low serum YKL-40 patients was statistically significant (p = 0.031) (Table 3).

**Table 3:** Response to chemotherapy and serum YKL-40 levels.

Clinical response	All patients	YKL-40 (high) (%)	YKL-40 (low) (%)	*p*-value
CR	32	14 (43.8)	18 (56.2)	
PR	33	24 (72.7)	9 (27.3)	
SD	35	28 (80.0)	7 (20.0)	
PD	20	15 (75.0)	5 (25.0)	
Response (CR+PR)	65	38 (58.5)	27 (41.5)	0.031*
Non-response (SD+PD)	55	43 (78.2)	12 (21.8)	

CR, complete response; PR, partial response; SD, stable disease; PD, progressive disease.

*Significant difference.

Pre-chemotherapy serum YKL-40 of patients who were sensitive to chemotherapy were significantly lower than that of non-responders (*p* = 0.004) (Figure 3B). Serum YKL-40 of responders after chemotherapy were significantly decreased (*p* = 0.029), while YKL-40 of non-responders did not change significantly (*p* = 0.256) (Figure 3C) (Table 4).

**Table 4:** Characteristics and serum YKL-40 distribution of SCLC patients with pre-and post- chemotherapy.

Therapeutic efficacy	N	pre-chemotherapy	post-chemotherapy	*p*-value
Response (CR+PR)	65	65.56±27.20	56.54±18.12	0.029*
Non-response (SD+PD)	55	79.75±25.99	73.71±29.41	0.256
Total	120	72.06±27.48	64.41±25.47	0.026*

CR, complete response; PR, partial response; SD, stable disease; PD, progressive disease.

*Significant difference.

### Association of Serum YKL-40 Levels with Survival

**Figure 4:**
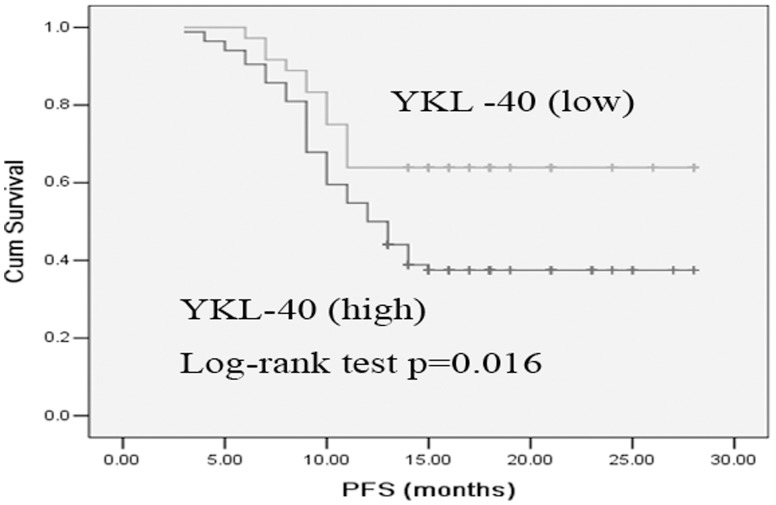
Kaplan-Meier curve comparing PFS of SCLC patients with high serum YKL-40 versus those with low serum YKL-40. Log-rank test determined that the PFS in low serum YKL-40 group was significantly longer than those of the high serum YKL-40 group (*p* = 0.016).

**Figure 5:**
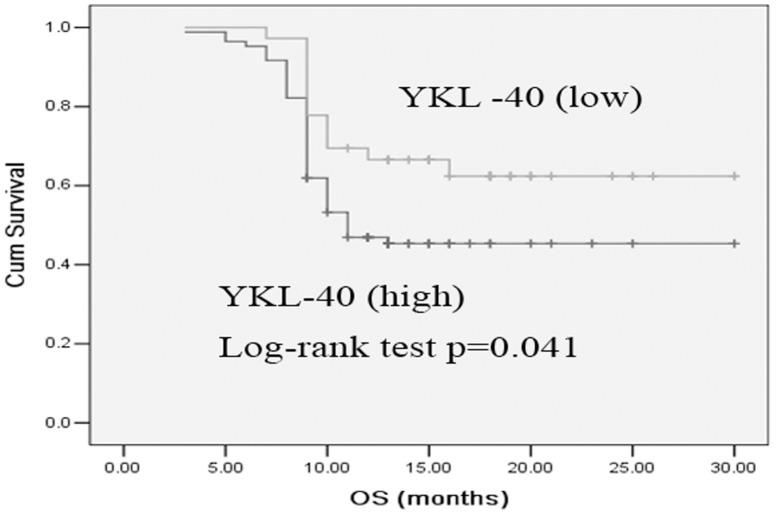
Kaplan-Meier curve comparing OS of SCLC patients with high serum YKL-40 versus those with low serum YKL-40. The Log-rank test showed that the survival times were significantly longer in patients with low serum YKL-40 than those of high serum YKL-40 (*p* = 0.041).

Low serum YKL-40 patients had significantly longer PFS and OS than high serum YKL-40 patients (Figure 4 and 5). The median PFS and OS were 6 and 10 months for high serum YKL-40 patients compared with 9 and 18 months for low serum YKL-40 patients, respectively. The 1-year survival rate was 40.7% in the 81 cases with high serumYKL-40, and 51.3% in the 39 cases with low serum YKL-40, a statistically significant difference (*p* = 0.041).

In univariate Cox analysis, the pre-chemotherapy serum YKL-40 level (dichotomized as >65.7 ng/mL vs ≤65.7 ng/mL) was associated with PFS (HR = 1.74, *p* = 0.033) and OS (HR = 1.33, *p* = 0.001) (Table 5 and Table 6). Univariate analysis of prognostic significance also showed that disease stage was significantly associated with poor survival. However, no association with the patient's prognosis was noted for age, gender, smoking, performance status and chemotherapy regimen.

**Table 5:** Univariate and multivariate Cox regression analysis of variables with PFS.

Variables	Univariate		Multivariate	
	HR (95% CI)	*p*-value	HR (95% CI)	*p*-value
Age (<60 *vs.* ≥60)	1.21 (0.72–2.02)	0.475		
Gender (female *vs.* male)	0.48 (0.22–1.04)	0.064		
Disease stage (limited *vs.* extended)	3.21 (1.15–8.93)	0.025*	2.34 (1.02–5.36)	0.046*
Smoking status (smoker *vs.* nonsmoker)	0.99 (0.96–1.02)	0.474		
Performance status (0, 1 *vs.* 2, 3)	1.70 (0.93–3.11)	0.082		
Chemotherapy regimen (EP *vs*. IP)	1.92 (0.69–1.983)	0.673		
YKL-40 (high *vs.* low)	1.74 (1.05-2.88)	0.033*	1.12 (1.01–1.23)	0.029*

HR, hazard ratio; CI, confidence interval; EP, cisplatin with etoposide; IP, cisplatin with irinotecan.

*Significant difference.

**Table 6:** Univariate and multivariate Cox regression analysis of variables with OS.

Variables	Univariate		Multivariate	
	HR (95% CI)	p-value	HR (95% CI)	*p*-value
Age (<60 *vs.* ≥60)	0.98 (0.43–2.23)	0.957		
Gender(female *vs.* male)	0.72 (0.51–1.02)	0.065		
Disease stage (Limited *vs.* Extended)	3.16 (1.36–7.40)	0.007*	2.13 (1.32–3.44)	0.002*
Smoking status (smoker *vs.* nonsmoker)	0.99 (0.97–1.01)	0.141		
Performance status (0, 1 *vs.* 2, 3)	1.09 (0.97–1.22)	0.089		
Chemotherapy regimen (EP *vs*. IP)	1.37 (0.65–2.86)	0.408		
YKL-40 (high *vs.* low)	1.33 (0.65–2.21)	0.001*	1.84 (1.08–3.15)	0.025*

HR, hazard ratio; CI, confidence interval; EP, cisplatin with etoposide; IP, cisplatin with irinotecan.

*Significant difference.

In multivariate Cox analysis, the pre-chemotherapy serum level of YKL-40 was independent prognostic factor for OS and PFS in the patients with SCLC (Table 5 and Table 6).

## Discussion

YKL-40 is a chitinase-like protein. The biologic functions of YKL-40 in cancer cells are unknown, and very few studies have evaluated the functional role of YKL-40 expression in cancer cells. It has been suggested that YKL-40 plays a role in the proliferation and differentiation of malignant cells, protects the cells from undergoing apoptosis, stimulates angiogenesis, has an effect on extracellular tissue remodeling, and stimulates fibroblast activity or proliferation surrounding the cancer cells [23], [24].

The objective of the current investigation was to study the association between the pre-chemotherapy serum YKL-40 level and chemotherapy response and clinical outcomes of patients with SCLC. In the present study, serum YKL-40 levels were higher in SCLC patients in line with the levels reported in most other types of cancer [25]–[27]. Furthermore, with a cut-off value of 65.7 ng/mL, YKL-40 had a sensitivity of 67.5% and a specificity of 95.0% for the prediction of SCLC, making it a potential adjunctive tool for diagnosis of SCLC. Importantly, the serum levels of YKL-40 were significantly correlated with disease stage, while there were no difference with age, gender, smoking status and performance status.

Reports regarding the association of YKL-40 expression with response to chemotherapy are few and varied. Gronlund et al reported that high serum levels of YKL-40 were associated with increased risk of second-line chemoresistance in patients with ovarian cancer [28]. However, Thöm et al found that the pretreatment serum YKL-40 level had no impact on response to chemotherapy in non-small cell lung cancer (NSCLC) [20]. Our study clearly showed that high serum YKL-40 patients response to chemotherapy were poorer than those of low serum YKL-40 patients. These results probably reflect different mechanisms of carcinogenesis between SCLC and NSCLC. Additionally, we observed that the pre-chemotherapy serum YKL-40 levels of the responder groups which were sensitive to chemotherapy were significantly lower than those of the non-responder groups which were resistant to chemotherapy. Moreover, serum YKL-40 levels were significantly decreased after chemotherapy, especially in the positive response group. To date, the mechanism of YKL-40 involved in resistance to chemotherapy is currently unclear.

Many studies have shown that serum YKL-40 was an important factor to predict the prognosis of several types of cancer [11]–[17]. Recently, Choi et al reported that high pretreatment serum YKL-40 level in patients with NSCLC was an independent prognostic variable of poor prognosis [18]. In SCLC patients, a high serum YKL-40 at the time of diagnosis and before chemotherapy was independent of prognostic variables for survival within the first 6 months and independent of age, performance status, and serum lactate dehydrogenase [19]. In our study, we have demonstrated that serum YKL-40 level in patients with SCLC was related to their very poor prognosis, as illustrated by the short PFS and OS in SCLC patients that had the highest serum YKL-40 levels. It is noteworthy that a high serum YKL-40 level was an independent prognostic biomarker of poor survival in this patient population. The mechanism involved in the association between YKL-40 and a poor prognosis is poorly understood. Previous studies suggested that poor survival in cancer patients with elevated YKL-40 might be attributed to the promotion of angiogenesis [17], [29].

In conclusion, the serum level of YKL-40 in SCLC patients was significantly higher than healthy controls. Serum YKL-40 in the positive chemotherapeutic response group was significantly lower after chemotherapy. The results not only further support that serum YKL-40 is related to chemotherapy response, but also provide clues that YKL-40 possess the potential to be a serum diagnostic and prognostic marker for SCLC. Moreover, further work is underway to both replicate these findings in a study with a larger cohort in lung cancer.

## References

[1] Chen Z, Wang T, Cai L, Su C, Zhong B, et al (2012) Clinicopathological significance of non-small cell lung cancer with high prevalence of Oct-4 tumor cells. J Exp Clin Cancer Res 31: 10. 22300949 10.1186/1756-9966-31-10 PMC3287152

[2] Maddison P, Thorpe A, Silcocks P, Robertson JF, Chapman CJ (2010) Autoimmunity to SOX2, clinical phenotype and survival in patients with small-cell lung cancer. Lung Cancer 70: 335–339. 20371131 10.1016/j.lungcan.2010.03.002

[3] J Barata F, Costa AF (2007) Small cell lung cancer-state of the art and future perspectives. Rev Port Pneumol 13: 587–604. 17898914 10.1016/s0873-2159(15)30365-2

[4] Govindan R, Page N, Morgensztern D, Read W, Tierney R, et al (2006) Changing epidemiology of small-cell lung cancer in the United States over the last 30 years: analysis of the surveillance, epidemiologic, and end results database. J Clin Oncol 24: 4539–4544. 17008692 10.1200/JCO.2005.04.4859

[5] Free CM, Ellis M, Beggs L, Beggs D, Morgan SA, et al (2007) Lung cancer outcomes at a UK cancer unit between 1998-2001. Lung Cancer 57: 222–228. 17442450 10.1016/j.lungcan.2007.03.006

[6] Okamura K, Takayama K, Izumi M, Harada T, Furuyama K, et al (2013) Diagnostic value of CEA and CYFRA 21-1 tumor markers in primary lung cancer. Lung Cancer 80: 45–49. 23352032 10.1016/j.lungcan.2013.01.002

[7] Tufman A, Huber RM (2010) Biological markers in lung cancer: A clinician's perspective. Cancer Biomark 6: 123–135. 20660959 10.3233/CBM-2009-0124 PMC12922859

[8] Grunnet M, Sorensen JB (2012) Carcinoembryonic antigen (CEA) as tumor marker in lung cancer. Lung Cancer 76: 138–143. 22153832 10.1016/j.lungcan.2011.11.012

[9] Johansen JS, Jensen BV, Roslind A, Nielsen D, Price PA (2006) Serum YKL-40, a new prognostic biomarker in cancer patients? Cancer Epidemiol Biomarkers Prev 15: 194–202. 16492905 10.1158/1055-9965.EPI-05-0011

[10] Bi J, Lau SH, Lv ZL, Xie D, Li W, et al (2009) Overexpression of YKL-40 is an independent prognostic marker in gastric cancer. Hum Pathol 40: 1790–1797. 19765801 10.1016/j.humpath.2009.07.005

[11] Schmidt H, Johansen JS, Sjoegren P, Christensen IJ, Sorensen BS, et al (2006) Serum YKL-40 predicts relapse-free and overall survival in patients with American Joint Committee on Cancer stage I and II melanoma. J Clin Oncol 24: 798–804. 16391295 10.1200/JCO.2005.03.7960

[12] Yamac D, Ozturk B, Coskun U, Tekin E, Sancak B, et al (2008) Serum YKL-40 levels as a prognostic factor in patients with locally advanced breast cancer. Adv Ther 25: 801–809. 18670741 10.1007/s12325-008-0082-2

[13] Høgdall EV, Ringsholt M, Høgdall CK, Christensen IJ, Johansen JS, et al (2009) YKL-40 tissue expression and plasma levels in patients with ovarian cancer. BMC Cancer 9: 8. 19134206 10.1186/1471-2407-9-8 PMC2645422

[14] Dupont J, Tanwar MK, Thaler HT, Fleisher M, Kauff N, et al (2004) Early detection and prognosis of ovarian cancer using serum YKL-40. J Clin Oncol 22: 3330–3339. 15310777 10.1200/JCO.2004.09.112

[15] Jensen BV, Johansen JS, Price PA (2003) High levels of serum HER-2/neu and YKL-40 independently reflect aggressiveness of metastatic breast cancer. Clin Cancer Res 9: 4423–4434. 14555515

[16] Wang D, Zhai B, Hu F, Liu C, Zhao J, et al (2012) High YKL-40 serum concentration is correlated with prognosis of Chinese patients with breast cancer. PLoS One 7: e51127. 23227243 10.1371/journal.pone.0051127 PMC3515550

[17] Saidi A, Javerzat S, Bellahcène A, De Vos J, Bello L, et al (2008) Experimental anti-angiogenesis causes upregulation of genes associated with poor survival in glioblastoma. Int J Cancer 122: 2187–2198. 18092325 10.1002/ijc.23313

[18] Choi IK, Kim YH, Kim JS, Seo JH (2010) High serum YKL-40 is a poor prognostic marker in patients with advanced non-small cell lung cancer. Acta Oncol 49: 861–864. 20553098 10.3109/02841861003631503

[19] Johansen JS, Drivsholm L, Price PA, Christensen IJ (2004) High serum YKL-40 level in patients with small cell lung cancer is related to early death. Lung Cancer 46: 333–340. 15541818 10.1016/j.lungcan.2004.05.010

[20] Thöm I, Andritzky B, Schuch G, Burkholder I, Edler L, et al (2010) Elevated pretreatment serum concentration of YKL-40-An independent prognostic biomarker for poor survival in patients with metastatic nonsmall cell lung cancer. Cancer 116: 4114–4121. 20564116 10.1002/cncr.25196

[21] van Meerbeeck JP, Fennell DA, De Ruysscher DK (2011) Small-cell lung cancer. Lancet 378: 1741–1755. 21565397 10.1016/S0140-6736(11)60165-7

[22] Therasse P, Arbuck SG, Eisenhauer EA, Wanders J, Kaplan RS, et al (2000) New guidelines to evaluate the response to treatment in solid tumors. European Organization for Research and Treatment of Cancer, National Cancer Institute of the United States, National Cancer Institute of Canada. J Natl Cancer Inst 92: 205–216. 10655437 10.1093/jnci/92.3.205

[23] Lee CG, Hartl D, Lee GR, Koller B, Matsuura H, et al (2009) Role of breast regression protein 39 (BRP-39)/chitinase 3-like-1 in Th2 and IL-13- induced tissue responses and apoptosis. J Exp Med 206: 1149–1166. 19414556 10.1084/jem.20081271 PMC2715037

[24] Shao R, Hamel K, Petersen L, Cao QJ, Arenas RB, et al (2009) YKL-40, a secreted glycoprotein, promotes tumor angiogenesis. Oncogene 28: 4456–4468. 19767768 10.1038/onc.2009.292 PMC2795793

[25] Zou L, He X, Zhang JW (2010) The efficacy of YKL-40 and CA125 as biomarkers for epithelial ovarian cancer. Braz J Med Biol Res 43: 1232–1238. 21103788 10.1590/s0100-879x2010007500133

[26] Mitsuhashi A, Matsui H, Usui H, Nagai Y, Tate S, et al (2009) Serum YKL-40 as a marker for cervical adenocarcinoma. Ann Oncol 20: 71–77. 10.1093/annonc/mdn552 18723551

[27] Schultz NA, Christensen IJ, Werner J, Giese N, Jensen BV, et al (2013) Diagnostic and prognostic impact of circulating YKL-40, IL-6, and CA 19.9 in patients with pancreatic cancer. PLoS One 8: e67059. 23840582 10.1371/journal.pone.0067059 PMC3694124

[28] Gronlund B, Høgdall EV, Christensen IJ, Johansen JS, Nørgaard-Pedersen B, et al (2006) Pre-treatment prediction of chemoresistance in second-line chemotherapy of ovarian carcinoma: value of serological tumor marker determination (tetranectin, YKL-40, CASA, CA 125). Int J Biol Markers 21: 141–148. 17013795 10.1177/172460080602100302

[29] Mylin AK, Andersen NF, Johansen JS, Abildgaard N, Heickendorff L, et al (2009) Serum YKL-40 and bone marrow angiogenesis in multiple myeloma. Int J Cancer 124: 1492–1494. 19089918 10.1002/ijc.24110

